# Exploring the Potential of *Moringa oleifera* in Managing Bone Loss: Insights from Preclinical Studies

**DOI:** 10.7150/ijms.103241

**Published:** 2025-01-21

**Authors:** Haryati Ahmad Hairi, Rusdiah Ruzanna Jusoh, Muhammad Zulfiqah Sadikan, Wan Nuraini Wan Hasan, Ahmad Nazrun Shuid

**Affiliations:** 1 Department of Biochemistry, Faculty of Medicine, Manipal University College Malaysia, Bukit Baru, 75150, Melaka, Malaysia.; 2 Department of Pharmacology, Faculty of Medicine, Manipal University College Malaysia, Bukit Baru, 75150, Melaka, Malaysia.; 3 Faculty of Bioeconomics, Food & Health Science, University of Geomatika Malaysia, Setiawangsa, 54200, Kuala Lumpur, Malaysia.; 4 Department of Pharmacology, Faculty of Medicine, Universiti Teknologi Mara (UITM), Jalan Hospital, 47000, Sungai Buloh, Selangor, Malaysia.

**Keywords:** *Moringa oleifera*, osteoporosis, bone remodelling, *in vitro*, *in vivo*, *ex vivo*

## Abstract

*Moringa oleifera* (MO) is renowned for its remarkable medicinal uses, supported by claims across various cultures and growing scientific evidence. Preclinical experimental evidence indicated that MO may effectively reduce bone loss and promote bone remodelling through its effects on osteoclasts and osteoblasts. *In vivo* studies demonstrated that MO enhances critical aspects of bone health, such as bone volume, trabecular thickness and overall bone density. Furthermore, MO positively influenced bone biomarkers including alkaline phosphatase and procollagen type 1 N-terminal propeptide, reflecting improved bone formation. Additionally, *in vitro* and *ex vivo* studies revealed that MO boosted bone regeneration, stimulated osteoblast activity and reduced inflammation. In terms of mechanisms, MO may modulate signalling pathways related to bone metabolism, such as BMP2, PI3K/Akt/FOXO1, p38α/MAPK14 and RANKL/RANK//OPG pathways. This evidence provides a strong foundation for future clinical research and potential therapeutic applications in managing and preventing bone loss conditions.

## 1. Introduction

Bone remodelling involves the constant regeneration and replacement of bone tissues, which occurs in two separate phases: bone formation and bone resorption. During bone formation, osteoblasts (bone-forming cells) fill the bone cavities with new tissue. While, during resorption, osteoclasts (bone-resorbing cells) disintegrate bone tissue. Under normal and healthy conditions, bone resorption and formation occur in a dynamic and balanced way, with old tissues constantly replaced by new ones 1. Calcium which is vital for bone health is primarily stored in bone. Calcium phosphate and mineralised collagen serve as structural supporting components for bone tissue. When blood calcium levels drop, various physiological responses contribute to maintaining calcium homeostasis. This includes activation of parathyroid hormone, which causes release of bone calcium, increased intestinal calcium absorption and increased renal calcium reabsorption. Traditionally, serum and urine calcium levels are employed as indications of mineral balance in the body. Meanwhile, excessive urine calcium excretion is linked to bone loss and osteoporosis 2.

Several clinical tests are now available for assessing bone health and evaluating therapy responses. Most of these indicators are also applicable to *in vitro* and *in vivo* experimental research. Bone mineral density (BMD), bone microarchitecture and biochemical markers serve as indicators of bone remodelling. In the clinical setting, bone diseases are typically evaluated using dual-energy X-ray absorptiometry (DXA), which measure bone mineral density. A T-score is created to indicate deviation of the measured BMD from the reference values in terms of standard deviation (SD). A T-score of -1 to -2.5 suggests poor bone mass or osteopenia, while a value below -2.5 indicates osteoporosis 3. Bone microarchitecture can be evaluated using quantitative computed tomography (qCT). Deterioration of bone architecture resulted from bone loss, as demonstrated by decreased trabeculae number, increased inter-trabecular distances, loose connectivity of the trabecular meshwork, reduction of cortical bone thickness and increased porosity 3.

In recent years, cellular components of bone tissue have been widely employed as biomarkers for measuring and monitoring bone turnover and bone loss. Novel markers representing osteoblast or osteoclast metabolic activity can be measured in blood or urine to provide a quantitative estimate of bone remodelling status. Information on bone remodelling status can be used to predict pathological changes or the likelihood of developing certain bone diseases. These biomarkers have been employed mostly to evaluate the efficacy of antiresorptive and anabolic medications used to treat bone diseases and compliance with these medication therapies. They may also be used to enhance fragility fracture risk evaluation 4, 5.

Bone defects can negatively impact a patient's quality of life, and treatments are becoming increasingly expensive. As the population ages, they are more likely to experience bone disorders like pain, fractures, osteoporosis, infections, tumours, rheumatic diseases and oral/maxillofacial pathologies. When bone resorption outpaces bone synthesis, bone tissues are lost, resulting in osteoporosis. Osteoporosis is a prevalent bone disease marked by decreasing bone mass and microstructure alterations. Patients with osteoporosis should regularly calculate their fracture risk as they are prone to fractures. Bone fractures from injury, infection or inflammation can heal through cell proliferation and regeneration 6. Certain plants contain phytocompounds that can enhance bone repair and prevent bone loss by inhibiting osteoclast cells recruitment, promoting osteoblast growth and lowering inflammation without the negative side effects associated with allopathic treatments 7.

*Moringa oleifera* (MO), commonly known as the 'drumstick tree' or 'horse radish tree,' is also referred to by various other names, including kelor, marango, mlonge, moonga, mulangay, nebeday, saijhan, sajna and ben oil tree 8. MO is a unique plant with numerous edible parts, each offering distinct biological activities. Its leaves, fruit pods, seeds, flowers and roots contribute to diverse range of potential health benefits. The leaves of MO are rich in sulphur-containing amino acids, β-carotene, L-ascorbic acid, calcium, potassium, iron and fibre, surpassing the levels found in carrots, oranges, bananas and spinach 9. MO seed oil primarily contains campesterol, stigmasterol, β-sitosterol, avenasterol and clerosterol, with trace amounts of 24-methylenecholesterol, campestanol, stigmastanol, and 28-isoavenasterol 10. Meanwhile, MO leaves and pods are rich in ascorbic acid, retinol, riboflavin, nicotinic acid, pyridoxine, α-tocopherol, folic acid, β-carotene, estrogenic substances, β-sitosterol, iron, calcium, phosphorus, copper, protein and essential amino acids such as methionine, cystine, tryptophan and lysine, making them ideal dietary supplements 11. Furthermore, laboratory studies demonstrated that mice fed with MO have elevated serum levels of calcium, proteins, phosphorus and antioxidants, while showing reduced levels of glucose, triglycerides and cholesterol 12, 13. MO stands out as an affordable source of nutrition that can potentially alleviate malnutrition. It is especially beneficial for children who lack access to breast milk and exhibit signs of nutrient deficiencies. Intriguingly, MO also provides affordable nutrition and has the potential to combat malnutrition. MO contains lactagogues, which are substances commonly prescribed to breastfeeding mothers to help increase milk supply. These lactagogues, composed of phytosterols, act as precursors to hormones essential for reproductive growth. In addition, MO contains phytosterols such as stigmasterol, sitosterol and campesterol, which support estrogen synthesis. This, in turn, promotes the growth of mammary gland ducts and enhances milk production 14.

Experiments on MO extract revealed its remarkable osteoprotective impact and ability to enhance osteogenesis. Some of MO's therapeutic benefits are directly related to its osteoprotective activities, as addressed further below. MO, which has strong antioxidant and anti-inflammatory properties, can counteract the defects in osteoblastogenesis and osteoclastogenesis-induced by osteoporosis. This review article explores the preclinical experimental evidence regarding MO's efficacy in reducing bone loss.

## 2. Potential Effects of MO in Treating Bone Loss: *In Vivo* Studies

This review was based on data obtained from PubMed, Google scholar and EBSCOhost Medline databases from their inception to June 2024. There were 11 published *in vivo* studies that investigated the potential effects of MO on bones (Table 1). In these studies, MO was extracted either from the leaves, seeds or roots. However, some studies did not indicate the dose/concentration of MO used in their studies. Kusolrat and Kupittayanant 16 conducted a 6-week trial using 0.25 mL/100 g bodyweight/day of MO seed oil. Both ovariectomised (OVX) rats treated with MO from the preventative and recovery investigations showed significantly lower levels of serum alkaline phosphatase (ALP) but higher serum calcium levels compared to OVX control rats. The urine calcium and phosphorus excretion were lower than OVX control rats. The increased bone ALP activity associated with ovariectomy was due to high bone turnover rate, as seen by accelerated bone formation and resorption, which may lead to osteoporosis 15. These bone marker patterns were also consistent with many prior studies, which found that vegetable oils rich in omega-3 and omega-6 polyunsaturated fatty promoted bone formation 16. On the other hand, Kusolrat and Kupittayanant reported that OVX rats given MO seed oil have lower total cholesterol and low-density lipoprotein cholesterol (LDL-C) levels, as well as higher high-density lipoprotein cholesterol (HDL-C) level 15. These hypocholesterolemic and anti-osteoporotic effects in OVX rats were consistent with other studies using dietary soybean, flaxseed and sesame oils 17-19.

Hu *et al.* 21 found that estradiol (E2) and MO had comparable efficacy in enhancing bone metabolism, microstructure and density in OVX rats. In their study, MO treatment decreased the number of osteoclasts and bone resorption indicators, which was similar to Kusolrat and Kupittayanant's study, where MO treatment enhanced serum calcium levels, potentially by reducing bone resorption. Hu *et al.* al also reported that MO treatment increased procollagen I n-terminal propeptide (PINP) and decreased bone ALP, demonstrating its osteogenic properties. The results showed that MO intervention increased bone formation, decreased bone resorption and slowed bone turnover. In comparison to the OVX group, the trabecular bone microstructure showed significantly higher levels of bone volume (BV/TV), trabecular thickness (Tb.Th), trabecular separation (Tb.Sp), trabecular number (Tb.N) and connectivity density (Conn.D) in the estradiol (E2) and MO groups. Histopathological study revealed trabecular damage (thinning, fracture, reduced area and wide trabecular spacing) apart from an abundance of fat vacuoles in the OVX group. E2 and MO treatments restored trabecular bone integrity to normal levels while reducing fat vacuoles in the OVX group 20. The data indicated that removing the ovaries decreased bone mass and destroyed bone microstructure, whereas estradiol and MO therapies may alleviate these effects.

Intriguingly, Hu *et al.* also examined gut flora composition, intestinal permeability and key signalling markers in the mitogen-activated protein kinase (MAPK) pathway to understand MO's osteoprotective mechanisms. Novel gut flora-targeting therapies like probiotics and prebiotics might be efficacious at preventing bone loss 20. Other studies demonstrated that supplementation with beneficial bacteria such as lactobacilli may offer protection against osteoporosis. Reductions in both the *Firmicutes/Bacteroidete*s ratio and *Lactobacillus* abundance are characteristics of the gut microbiota in OVX rats 21, 22. The study supported previous findings that MO could improve osteoporosis by increasing the *Firmicutes/ Bacteroidetes* ratio and *Lactobacillus* abundance. The phylum *Fusobacteria, Elusimicrobia and genus Oscillospira* were found to have a favourable correlation with BMD. Microorganisms were seen to be more abundant in the normal control group than in the OVX group. Thus, these bacterial taxa may have anti-osteoporotic properties. Subsequently, Hu *et al.* used Cytoscape v. 3.7.2 to create a target pathway network map in identifying the core pathways and targets of MO in osteoporosis treatment. According to the analysis, MAPK signalling may be the core pathway through which MO increase extracellular signal-regulated kinase (ERK) expression and exerted their anti-osteoporosis effect. Other fascinating findings include *Lactobacillus'* ability to convert dietary L-histidine into immunomodulatory histamine, which raises cAMP levels and suppresses ERK activation 20. Macrophages play an important function in bone development. *Lactobacillus* stimulates the immunological response of bone marrow-derived macrophages by activating MAPKs 23. In addition, MO contains myricetin, quercetin and kaempferol, which can ameliorate osteoporosis. Other research has shown that myricetin reduces glucocorticoid-induced osteoporosis by modulating ERK signalling, enhancing femoral histology and increasing osteogenic differentiation and matrix mineralisation 24.

Diabetes mellitus is known to be correlated with an increased risk of osteoporosis. Streptozotocin is primarily employed as a chemical to create type 1 diabetes mellitus (T1DM) animal model. Ovariectomised rat is a classic animal model of oestrogen deficiency-induced osteoporosis. Both oestrogen deprivation and T1DM can cause osteoporosis 25. In an *in vivo* study by Patel *et al.* 27, OVX and streptozotocin-induced osteoporosis model in rats was utilised to evaluate MO's preventive effects on bone loss caused by ovariectomy paired with diabetes. Patel *et al.* employed three separate components: leaves, fruits, and flowers, with findings showing all MO treatments reduced glucose levels in STZ-OVX rats. Among the three components, fruit extract was found to be very effective in lowering the raised glucose levels as well as TRAcP, the osteoclastic bone marker, in STZ-OVX rats. On the other hand, it caused a rise in ALP, the ostoblastic marker, in STZ-OVX rats 26. The study provided evidence that MO fruit extracts play a major role in the prevention of bone loss in STZ-OVX mice.

Glucocorticoids (GCs) are the leading iatrogenic cause of secondary osteoporosis, with fracture rates increased by up to 75% within the first three months of treatment. GCs increase the production of macrophage colony-stimulating factor (M-CSF) and receptor activator of nuclear factor kappa-beta (NF-κB) ligand RANKL and decrease the production of osteoprotegerin (OPG) by osteoblastic cells and osteocytes. These led to increase number and activity of osteoclasts, with direct effects on bone resorption 27. In the study by Soliman *et al.*, osteoporosis was induced in the jawbone of Albino rats by daily intraperitoneal injection of 200μg/100g body weight of dexamethasone for 30 days. Bone density scan (DEXA) analysis confirmed osteoporosis of the upper jawbone (mandible). However, DEXA analysis showed a significant increase in BMD in the MO leaves-treated group compared to the steroid induced osteoporotic group. Based on Real-time PCR analysis, the MO leaves group also had significantly lower RANKL and higher OPG gene expression levels. Histological evaluation of the MO leaves group revealed significant healing of the mandible microanatomy. Histomorphometric analysis indicated a substantial increase in the percentage bone area in the mandible of the MO leaves group 28.

A similar study from Habib *et al.* employed a glucocorticoid-induced osteoporosis rat model, in which 100 mg prednisone acetate per kg meal was used as a glucocorticoid source to induce osteoporosis for two weeks. In the study, diets containing dried MO leaves (2.5%), moringa seeds (2.5%) and a mixture of both (2.5%) were fed to rats. Supplementation with MO leaves, seeds, or combinations significantly enhanced blood calcium and phosphorus levels in osteoporotic rats. In osteoporotic rats, serum-free thyroxin (T4) levels increased significantly, while parathormone (PTH) levels decreased significantly. Supplementation with MO (leaves, seeds, or combination) significantly boosted serum T4 levels, while decreasing serum PTH levels. The parathyroid organ produces parathyroid hormone, which regulates bone digestion and calcium homeostasis, among other metabolic hormones. The study discovered that rats with osteoporosis had significantly higher levels of PTH compared to controls. This may stimulate bone resorption to increase serum calcium levels, which could lead to osteoporosis. Following MO supplementation, the rat femoral BMD was significantly higher compared to osteoporotic rats 29. A similar study conducted by Rawat *et al.* reported that MO supplementation ameliorated the steroid-induced reductions in BMD of the rat femur, serum calcium and ALP levels 30. These studies concluded that MO leaves, seeds and their combinations have effective anti-osteoporotic properties.

Long-term glucocorticoid use can result in femoral head necrosis, which causes persistent deformity, fractures and aberrant bone regeneration 31. In the study from Zhang *et al.*, the chemical structure of purified polysaccharide from the MO plant was analysed using Fourier transform-infrared spectroscopy (FT-IR), methylation and nuclear magnetic resonance spectroscopy (NMR) methods. Osteoporosis was induced in rats by giving 0.2 g/kg injection of dexamethasone every four days. The rats also received daily oral gavages of MO polysaccharide at 6 g/kg (an average of 1.32 g/rat). The structural analysis revealed that MO polysaccharide contains D-galactitol, L-arabinitol and L-rhamnitol, as well as non-reduced L-rhamnopyranosyl, D-galactopyranosyl, L-arabinopyranosyl and D-galactopyranosyl. The study identified that rats given MO polysaccharide have improved bone volume, trabecular thickness, bone density and hexosamine concentration. Meanwhile, micro-CT scan results revealed that MO polysaccharides supplementation could counteract the bone-deleting effects of dexamethasone by maintaining the trabecular bone thickness. MO polysaccharide also increased the expressions of osteocalcin (OCN) and Runt-related transcription factor 2 (Runx2) genes, which could help to preserve bone volume and trabecular thickness parameters. High levels of hydroxyproline (a non-essential amino acid in collagen) and hexosamine (a component of mucopolysaccharide that reduces osteoblastic formation and bone reabsorption) in blood can indicate femoral bone necrosis 32. MO polysaccharides can enhance collagen and mucopolysaccharide synthesis by increasing the hexosamine-to-hydroxyproline ratio 33. In the study, it was discovered that ingestion of MO polysaccharides significantly increased the expression level of the COL-1 gene, which is particularly effective at healing damaged tissue. The findings suggest that MO polysaccharides could be used to control femoral head necrosis.

Tooth extraction is the process of removing a tooth from its alveolar bone socket, which can cause an inflammatory response and alveolar bone resorption in the buccolingual and apicocoronal areas of the edentulous ridge 34. Alveolar bone resorption begins with the binding of RANKL to its receptor, receptor activator of nuclear factor-kappa B (RANK), which is found on preosteoclasts 35. RANKL/RANK is a critical regulator of osteoclastogenesis. Proinflammatory cytokines including TNF-α, IL-1 and IL-6 can also influence the development of osteoclasts. Osteoprotegerin (OPG) protects bone from excessive resorption by binding to RANKL, preventing it from binding to RANK. RANKL and OPG bonds are the primary factors influencing bone mass and strength 36. One approach for preventing alveolar bone loss is socket preservation. It is a surgical treatment that preserves the alveolar bone and soft tissues to the greatest extent possible after tooth extraction 37. Demineralised freeze-dried bovine bone xenograft (DFDBBX) is a biocompatible and osteoinductive replacement material that can be utilised to preserve alveolar bone sockets 38. The use of DFDBBX and MO combination has received attention as it could lessen the inflammatory response following tooth extraction. Soekobagiono *et al.*, discovered that combining MO leaf extract with DFDBBX significantly reduced the level of RANKL expression in alveolar bone sockets of guinea pigs after tooth extraction 39. Using immunohistochemistry Djais *et al.*, found that combination of MO and Demineralization Freeze Dried Dentin Matrix (DFDDM) protected tooth extraction sockets against bone loss by increasing OPG and decreasing RANKL expressions 40. Furthermore, histological and histomorphometric analyses revealed that MO leaf extract significantly increased the surface area of bone and the number of osteoblasts in MO groups compared to control groups, demonstrating its positive effect on bone regeneration in critical-sized defects 41.

Bone healing is a very complex reformative process. Bone tissues commonly heal spontaneously but may be incomplete in complicated circumstances such as large bone defects. Bone healing can be divided into three phases: inflammatory phase, reparative phase and remodelling phase 42. Al-Azzawi and Al-Ghaban created a femur defect in albino rats to study the potential of MO and marine collagen (MC) in bone healing. Bone microarchitectures during healing was assessed using histological and histomorphometric analysis. Microscopic analysis of serial sections from the intervention location two weeks after treatment with MO extract revealed a considerable increase in osteoblasts compared to the control group. After 2- and 4 weeks, the group treated with MO showed increased osteoblast activity and decreased osteoclast activity, resulting in increased trabecular area and decreased bone marrow area compared to the control group. Intriguingly, after two weeks, the combination of MO and MC groups had more osteoblast cells and larger bone trabecular regions than either group alone. At 4 weeks, the trabecular bone area was significantly larger compared to two weeks, while bone trabeculae seemed thicker with regularly ordered osteocytes 43.

## 3. Potential Effects of MO in Treating Bone Loss: *In Vitro and Ex vivo* Studies

There were five *in vitro* and *ex vivo* studies which have assessed MO's osteoinductive potential in several cell types, including bone marrow mesenchymal stem cells (BMSCs), human osteosarcoma cell lines (SaOS2 and UMR106) and murine preosteoclast cell lines (RAW 264.7) (Table 2). *In vitro* studies were conducted to examine MO's effects on cell viability, proliferation and mineralisation, showing that MO can directly promote osteoblast development and differentiation. MO could stimulate cell proliferation, ALP activity, collagen synthesis and calcium deposition of osteoblast cells in a dose-dependent manner. Interestingly, MO leaves, fruits, seeds, flowers and roots have all been shown to possess osteoinductive properties.

Patel *et al.* investigated the osteoblastogenic potential of MO extracts from various parts (leaves, flowers and fruits) on SaOS2 osteoblast cell line. The MO extracts increased osteoblast proliferation from the dose of 50 μg/mL to 100 μg/mL. The study demonstrated that methanolic extracts of all MO parts promoted osteoblastogenesis. Flowers and fruit extracts were the most effective parts. The flower extract stimulated osteoblast cell proliferation, whereas the fruit extract increased ALP activity, hydroxyproline, calcium and collagen levels. Calcium is the primary component of bone mineral hydroxyapatite, while hydroxyproline is an essential amino acid for collagen production and bone matrix composition Although both extracts promoted bone formation, their mechanisms were slightly different. The flower extract increased osteoblast number, while the fruit extract promoted bone formation activity and mineralisation. 44. These findings indicated that MO extracts of various parts have positive impact on osteoblastic cell proliferation, activity, bone matrix formation and mineralization.

Using a comparable cell line model, Khan *et al.* discovered that in a cell viability assay, MO leaf extract promoted the growth activity of osteoblast cell line at the concentrations of 25 and 50 μg/mL. However, higher MO concentrations of 100 and 200 μg/mL reduced proliferation activity, probably adversely affected by ROS generation and chromatin condensation. Cell cycle analysis demonstrated that MO extracts at doses of 50 and 100 μg/mL halted cell division at the G2/M phase. The study also found that low doses of MO leaves extract increased the expression of osteogenic genes BMP2 and Runx2, while high doses decreased the expressions 45. BMP2 is a potent cytokine that promotes mesenchymal cell *in vitro* differentiation into osteoblasts and *in vivo* bone formation. BMP2 promotes osteogenesis by activating Smad signalling and regulating the transcription of osteogenic genes such as Runx2, type I collagen, osteocalcin and bone sialoprotein. Runx2 plays a crucial role in early osteogenesis by regulating bone production, late mineralisation and expression of critical bone matrix genes during osteoblast differentiation 46. The biphasic dose-response of osteoblast-like SaOS-2 cells growth activity towards MO extract suggested potential therapeutic and preventative applications in drug development.

Mesenchymal stem cells (MSCs) are stromal cells that can self-renew and differentiate into many lineages. MSC-based therapy has been proven to significantly increase tissue regeneration in pre-clinical and clinical trials 47. MSCs can differentiate into a specific set of cell lineages: adipogenesis, osteogenesis and chondrogenesis. It is crucial to search for new mediators that can boost MSCs' osteogenic differentiation capacity 48. The study by Marupanthorm and Kedpanyapong was the first to show that MO therapy could improve osteogenic differentiation of porcine bone MSCs (BMSCs), as indicated by enhanced ALP expression. Intriguingly, the study had used low concentrations of MO leaves ethanolic extract (100, 200 and 300 ng/ml). The optimal concentration of 100 ng/ml produced the most favourable results on osteogenesis 49. Following that, Liu *et al.* investigated the therapeutic effects of MO leaves extracts on rat BMSCs exposed to peroxidative damage by hydrogen peroxide (H_2_O_2_). The molecular mechanisms involving the PI3K/Akt/FoxO1 pathway were also investigated. It was found that this pathway was activated in response to peroxidative damage to promote cell survival 50. FoxO1 is a key regulator of oxidative stress and oxygen-free radical equilibrium during bone formation 51. The study found that oxidative stress caused by H_2_O_2_ reduced FoxO1, while MO leaves extract restored it. On top of that, the study demonstrated that FoxO1 played a crucial role in reducing oxidative damage in BMSCs undergoing osteogenic differentiation. MO leaves extract enhanced the osteogenic differentiation capacity of MSCs, suggesting its potential for bone regeneration and alternative medicine applications.

Osseointegration refers to a time-dependent healing process in which a stable, clinically asymptomatic attachment of artificial materials to bone is achieved and sustained under functional loading 52. In scientific term, osseointegration is bone ingrowth into a metal implant. The focus is on making the implant surface hydrophilic to improve osseointegration. Hydrophilic implant surfaces can help with bone integration and enhance osseointegration in the early stages of wound healing. Primary stability during installation, biological host bone and direct bone-to-implant contact (BIC) all contribute significantly to implant life and success 53-55. Histomorphometry at the light microscopic level has been utilised to determine the amount of bone apposition surrounding the implant. For instance, Pachimalla *et al.* conducted a study to create a hydrophilic gel from MO seed extract applied to the surface of a dental implant grown with human mesenchymal cells to improve bone-implant contact. Transmission electron microscopy (TEM) and histomorphometry analysis revealed that hydrophilic implant surfaces increased BIC and encouraged new bone formation. The addition of bone-proliferating components to these hydrogels would hasten osseointegration and strengthen bone structure 56. Surface features of implants, including topography, coatings and wettability, influence the behaviour of host osteoblasts and aid in bone formation during osseointegration. In particular, surface topography is essential for the initial adhesion and differentiation of osteoblasts on the implant surface and plays a significant role in long-term bone remodelling 52. Furthermore, there was no evidence of degeneration, necrosis, fibrosis or inflammation at the new bone-to-implant contact 56.

Periodontitis is a chronic condition characterised by the clinical loss of alveolar bone and degeneration of connective tissue. Previous studies have linked p38α MAPK signalling to osteogenesis and the expression of inflammatory cytokines in periodontitis 57, 58. Wang *et al.* have identified phenolic compounds in MO leaves extract using UPLC-ESI-MS/MS. Following that, the anti-periodontitis effects and mechanisms of MO were predicted through network pharmacology and molecular docking. Molecular docking analysis has identified p38α MAPK (MAPK14) pathway and the OPG/RANKL complex as potential targets for the anti-periodontitis effects. The *in vitro* study using RAW 264.7 pre-osteoclast cell lines demonstrated that MO significantly reduced inflammatory cytokines such as TNF-α, IL-1β, and IL-6, IL-1Ra and IL-10 and lowered OPG/RANKL ratio through MAPK14 inhibition. These MO effects halted the alveolar bone resorption by osteoclasts. In these findings, the anti-periodontitis effect might be attributed to the antioxidant properties of MO leaves extract 59.

## 4. Insight Molecular Mechanism of MO on Bone Remodelling

Mitogen-activated protein kinases (MAPKs) are essential dual-specificity protein kinases that participate in signalling pathways which control a variety of biological processes. MAPKs play a distinct role in stress response, cell proliferation, differentiation and survival. Moreover, they serve an important role in converting external signals into appropriate biological responses, which influence processes including inflammation, cell proliferation and death 60. p38 MAPK is one of the signalling pathways that plays a vital role in regulating the expressions of Runx2 and BMP2, which are essential for osteoblast development and bone formation.

This pathway affects Runx2 and BMP2 expressions through several mechanisms, including the phosphorylation of transcription factors, modulation of gene promoter activity and interactions with other signalling molecules. By regulating the expression of Runx2 and BMP2, p38 MAPK contributes significantly to bone growth, maintenance and repair 61.

Osteogenesis is the process of new bone formation, where transcription factors are crucial in regulating cell proliferation and differentiation. These transcription factors guide the development of osteoblasts, cells responsible for bone formation, ensuring proper bone growth and maturation 62. Runt-related transcription factor 2 (Runx2) is a crucial transcription factor governing the differentiation of mesenchymal stem cells into osteoblasts, which subsequently mature into osteocytes. Runx2 serves as a modulator capable of stimulating and inhibiting the differentiation of osteoblasts. For instance, in mice with a homozygous knockout of the Runx2 gene, bone formation was significantly impaired due to insufficient osteoblast differentiation and reduced expression of osteoblastic genes, including alkaline phosphatase (ALP) and osteocalcin (OC), among others 63. Therefore, it can be said that Runx2 is a key transcription factor and an early indicator of osteoblastogenesis.

As a member of the transforming growth factor-beta (TGF-β) superfamily, Bone morphogenetic proteins (BMPs) are crucial in bone formation and repair. BMP2 is a potent osteoblastogenic factor that stimulates the formation of osteoblasts by activating the Smad signalling pathway. This pathway involves phosphorylation of Smad proteins, complex formation with Smad4, as well as regulation of gene expressions related to osteoblast differentiation and bone formation. Smad1/5/8 proteins formed complex with Smad4 and translocated to the nucleus where they activate Runx2, resulting in increased expression of osteoblastogenic markers 64. Yoshikawa *et al.* discovered that BMP-2-induced RANKL mRNA expression was inhibited in primary osteoblasts lacking Smad1, leading to reduced osteoclast formation. In addition to Smad2 and Smad3, which mediate TGF-β signalling, as well as Smad1 and Smad5, which mediate BMP pathways, Smad4 plays a role in osteoclast formation by binding to phosphorylated R-Smads, acting as a Co-Smad in response to TGF-β/BMP stimulation 65. TGF-β/BMP signalling can be modulated by extracellular signal-regulated kinase (ERK), c-Jun N-terminal kinase (JNK), p38 MAPK, Ras homolog family member A (RhoA), phosphatidylinositol 3-kinase (PI3K) and protein kinase B (Akt), with effects depending on the specific cellular context 66.

p38 MAPK was reported to promote osteoclast development, maturation and bone resorption. Previous research on the role of p38 MAPK in osteoclastogenesis primarily involved *in vitro* studies using RAW264 cell line and primary bone marrow cells 67, 68. These studies revealed that p38 MAPK acted downstream of RANK following stimulation by RANKL, a key pro-resorptive cytokine essential for osteoclast formation. A complete inhibition of p38 MAPK effectively blocked osteoclastogenesis-induced by RANKL. Additionally, in osteoclast precursors, upon activation by TNF-α, p38 MAPK also operated downstream of the TNF receptor to support early osteoclast differentiation 69.

The differentiation of osteoclasts is primarily regulated RANKL/RANK pathway, where RANKL binds to its receptor RANK on osteoclast precursors, promoting their formation. Conversely, OPG, a soluble decoy receptor, inhibited this process by binding to RANKL and preventing it from interacting with RANK. RANKL also interacted with macrophage colony-stimulating factor (MCSF) to stimulate osteoclast development. These three components worked together to keep bone remodelling in equilibrium 70, 71. Disruption of this balance may lead to osteoporosis, which can be efficiently treated by blocking the RANKL/RANK pathway 72, 73. This was confirmed by the fact that OPG knockout mice suffered osteoporosis, demonstrating the relevance of OPG in controlling osteoclast development 74.

TNF-α, IL-1β and IL-6 are inflammatory cytokines that have impact on osteoclast activity, which is responsible for bone resorption. These cytokines influenced osteoclastogenesis (the production of osteoclasts) by interacting with RANKL, the key regulator of bone resorption process 75, 76. TNF-α promoted bone resorption by activating osteoclasts, and if uncontrolled may lead to bone deterioration. It also suppressed osteoblast function, which reduced bone growth. Elevated TNF-α levels from chronic inflammation or autoimmune diseases can impair bone density, worsen bone loss and increase the risk of osteoporosis 77. IL-1 is another cytokine which is important in inflammatory bone loss as it influences numerous elements of osteoclast biology, such as differentiation, multinucleation and survival. IL-1β plays a significant role in increasing osteoclast development. It accomplished this by boosting the level of RANKL, which is required for osteoclastogenesis 78. IL-1β and TNF-α promoted the production of osteoclasts by increasing RANKL expression in stromal cells, leading to differentiation of osteoclast precursors 79, 80. Furthermore, IL-1 can promote osteoclast development through pathways other than the RANKL/RANK pathway, particularly via the bone marrow-derived macrophages (BMMs) 81. When coupled with TNF-α, IL-1 activated osteoclasts to resorb bone, resulting in bone loss. Furthermore, IL-1 may impair fracture healing by inhibiting osteoblast migration, which impeded the repair process 82. IL-6 is another cytokine that has diverse effects on bone metabolism. It is best known as a pro-osteoclastic factor that promoted osteoclast activity. In mouse models with elevated IL-6 levels, an increase in osteoclasts and a decrease in bone trabecular volume could be seen, indicating enhanced bone resorption. IL-6 can induce osteoclast production through RANKL-dependent and independent pathways 83, 84. It can also directly or indirectly increase osteoclastogenesis by boosting RANKL synthesis by osteoblasts. This dual role highlighted IL-6's ability to promote bone loss via increased osteoclast activity.

Forkhead box class O (FoxO) proteins are evolutionarily conserved transcription factors that convert environmental signals into gene expression, regulating a variety of cellular processes including proliferation, differentiation, survival, oxidative stress, inflammation, apoptosis and ageing 85. FoxO proteins protect osteoblast progenitors from oxidative stress, which can damage proteins, lipids and DNA, potentially leading to cell death 86. Excess reactive oxygen species (ROS) contribute to oxidative stress. When FoxO1 is overexpressed, it enhances the production of these antioxidant enzymes, which helps to neutralise ROS and reduce oxidative stress within cells. Conversely, when FoxO1 is deleted, the protective effect against oxidative stress is lost, leading to increased ROS levels. This heightened oxidative stress can adversely affect the bone marrow, thus increasing osteoclast progenitors, which are precursor cells that develop into bone-resorbing osteoclasts 87. Thus, the loss of FoxO1 function can disrupt normal bone homeostasis by promoting the proliferation of osteoclast precursors and potentially contributing to bone loss.

Studies have shown that deleting FoxO1, FoxO3 and FoxO4 in mice can elevate oxidative stress and osteoblast apoptosis, as well as a decrease in the number of osteoblasts, bone formation rate and overall bone mass in cancellous and cortical bone regions 87, 88. This showed that FoxOs played an important role in protecting osteogenesis from oxidative stress. ROS such as H_2_O_2_ can activate FoxO targets by binding with β-catenin, a dual function protein, responsible for regulating and coordinating gene transcription and cell to cell adhesion 89.

The PI3K/AKT signalling pathway played a crucial role in regulating various cellular processes such as proliferation, differentiation, metabolism and survival 90. Findings from Liu *et al.* indicated that MO leaf extract activates PI3K, facilitating osteogenic differentiation in H_2_O_2_-damaged bone marrow stromal cells (BMSCs) 50. This activation may improve osteogenic differentiation (bone formation) of BMSCs injured by oxidative stress (H_2_O_2_). RNA sequencing analysis revealed that the PI3K/Akt pathway is involved in bone regeneration 91. Another study demonstrated that astragaloside promotes osteogenic differentiation of MC3T3-E1 cells by regulating the PI3K/Akt signaling pathway 92. Furthermore, an ex vivo study showed that exosomes derived from human-induced pluripotent stem cell-derived mesenchymal stem cells (hiPS-MSC-Exos), when combined with tricalcium phosphate, can repair critical-sized calvarial bone defects by activating the PI3K/Akt signaling pathway 93. FoxO1 is particularly affected by this pathway through PI3K/AKT-mediated phosphorylation. When AKT activity is inhibited, FoxO1 translocated to the nucleus and promoted the expression of pro-apoptotic genes such as Bax, caspase 3 and 9, leading to increased cell apoptosis 94, 95. In experimental conditions, dexamethasone treatment inhibited the PI3K/AKT signalling pathway by decreasing the levels of phosphorylated PI3K (p-PI3K) and phosphorylated AKT (p-AKT). This resulted in reduced phosphorylation of FoxO1 (p-FoxO1) and promoted FoxO1's movement into the nucleus. Conversely, the addition of insulin-like growth factor 1 (IGF-1) counteracted the effects of dexamethasone by enhancing p-PI3K, p-AKT and p-FOXO1 levels, while decreasing the total FOXO1 expression and its nuclear translocation. These findings demonstrate that dexamethasone regulated FOXO1 through the PI3K/AKT pathway, specifically by inducing FoxO1's nuclear entry and activating downstream apoptotic signalling pathways 95.

Remarkably, MO may affect several key signalling pathways related to bone metabolism, including the p38α/MAPK14, BMP2, RANKL/RANK/OPG and PI3K/Akt/FoxO1 pathways. These interactions suggested that MO could play a role in enhancing bone formation, regulating bone cell activity, managing inflammation and influencing bone resorption. These actions indicated that MO has potential therapeutic benefits for managing and preventing conditions related to bone loss. The evidence supporting its effects on these signalling pathways provided a promising foundation for future clinical research to explore its effectiveness and safety in bone health applications.

## 5. Bioactive Compounds of MO with Potential Osteoprotective Effects

Polyphenols are roughly divided into two types: phenolic acids (which have just one phenol ring) and flavonoids (which include many phenol rings). The MO tree contains several essential polyphenols, including phenolic acids and flavonoids, including tannins 96. The leaves of MO have been reported to have the highest total phenolic content, ranging from 2000 to 12,200 mg GAE/100 g. The most prevalent flavonoids found in the MO tree are kaempferol glycosides (including glucosides, malonyl glucosides, and rutinosides), as well as quercetin. Myricetin, epicatechin, and rutin are all present in small amounts 97.

Kaempferol (3,5,7-trihydroxy-2-(4-hydroxyphenyl)-4H-1-benzopyran-4-one) is a naturally occurring flavonoid that enhances the nutritional value of many fruits and vegetables. It is also found in various botanical plants that have long been used in traditional medicine, such as Ginkgo biloba and propolis 98. Kaempferol and its derivatives are known for their antioxidant, anti-inflammatory, and potential therapeutic qualities, making them valuable for both nutritional and medical applications. Preclinical investigations have shown that kaempferol has significant bone-protecting effects. In the review by Wong *et al.*, kaempferol supplementation has demonstrated bone-sparing effects in various models, including newborn rats, glucocorticoid-induced, ovariectomy-induced osteoporotic models, and bone fracture models. These bone-protective effects are achieved through multiple mechanisms: kaempferol inhibits adipogenesis, inflammation, oxidative stress, osteoclastic autophagy, and osteoblastic apoptosis, while promoting osteoblastic autophagy. The anti-osteoporotic effects of kaempferol are mediated by the regulation of key signaling pathways, including the estrogen receptor, bone morphogenetic protein-2 (BMP-2), nuclear factor-kappa B (NF-κB), mitogen-activated protein kinase (MAPK), and mammalian target of rapamycin (mTOR) pathways 99.

Quercetin is a broad group of naturally occurring flavonoid compounds found in plant-based diets. It is also a member of a class of plant-derived, nonsteroidal chemicals known as phytoestrogens 100. Quercetin has been found to improve bone metabolism by modulating a variety of cellular processes. It suppresses RANKL-mediated osteoclastogenesis, hence preventing excessive bone resorption. Furthermore, quercetin inhibits osteoblast apoptosis, oxidative stress, and the inflammatory response, all of which contribute to bone loss. On the other hand, quercetin stimulates osteogenesis, angiogenesis, and antioxidant expression, all of which are required for bone growth and repair. Furthermore, it promotes adipocyte and osteoclast apoptosis, which improves bone health by reducing fat cell growth and suppressing excessive bone resorption 101, 102.

Carotenoids are a broad collection of fat-soluble pigments that give different fruits, vegetables, fungi, bacteria, and algae their red, orange, and yellow colour. Carotenoids, including β-carotene, are precursors to vitamin A, making these pigments essential for human nutrition. Fresh Moringa (Moringa oleifera) leaves contain high levels of β-carotene, with quantities ranging from 6.6 to 17.4 mg per 100 g. This is significantly higher than the β-carotene level of other typical sources such as carrots, pumpkins, and apricots. Dried Moringa leaves contain significantly more β-carotene, with concentrations ranging from 23.31 to 39.6 mg per 100 g dry matter 103. This makes Moringa leaves a good source of this essential nutrient, especially when dried. Kulczyński *et al.* conducted a narrative review and found that carotenoids may improve bone health. Increased carotene intake and blood levels are consistently linked to improvements in critical bone health markers such as bone mineral density (BMD), alkaline phosphatase (ALP), C-terminal telopeptide (CTx), tartrate-resistant acid phosphatase (TRAP), and RANKL. Furthermore, carotenoids have been related to a lower risk of fracture, implying that they contribute to bone strength and integrity. These findings underscore carotenoids' potential involvement in improving overall bone health 104.

## 6. Safety Studies of MO

Safety studies on herbal medicines typically involved acute and subacute toxicity testing in laboratory animals. The acute toxicity study of methanol extract of MO revealed a high safety profile, as methanol extract of MO did not cause noticeable toxicity or mortality at the dose of 2000 mg/kg in any of the animals throughout the observation period. Haematological and biochemical parameters remained stable compared to the control group. These findings confirmed the safety of methanol extract of MO and supported its use as herbal medicine 105. However, a study by Oyagbemi found that rats administered methanol extract at doses of 200 and 400 mg/kg for 8 weeks exhibited significant increments (p < 0.05) in serum ALT, AST, BUN and creatinine levels, indicating potential hepatic and kidney damage 106. Meanwhile, other research found genotoxicity at supra-supplementation levels of 3000 mg/kg body weight, with intake remaining safe at levels of 1000 mg/kg or less 107.

The aqueous extract of MO leaves has also been found to adversely affect conception and pregnancy outcomes in rats. A dose of 58 mg/kg body weight of cold aqueous extract or 50 mg/kg body weight of hot aqueous extract administered before mating resulted in 100% and 83.3% infertility, respectively. The 58 mg/kg body weight dose was noted to be equivalent to 250 mg of the dry plant product. When rats were given these doses after mating, an 80% abortion rate was reported with the cold aqueous extract and a 50% abortion rate with the hot aqueous extract. In comparison, there were no abortions in the control group, which only received distilled water 108. In another study, Ajuogu *et al.* investigated the effects of adding 0 to 15 g/kg MO leaf powder in the diet on reproductive hormones and semen quality of rabbits after 12 weeks. In female rabbits, follicle-stimulating hormone (FSH) levels were lower with increasing MO powder doses compared to the control group. Conversely, male rabbits (bucks) displayed higher FSH levels with 15 g/kg MO powder compared to the control. Additionally, semen quality and sperm count improved with higher doses of MO powder. The study also revealed that while MO positively influenced buck fertility, it caused infertility in female rabbits 109.

MO is commercially accessible in capsule and powder forms, which is marketed for a variety of applications. Several research have investigated the clinical safety and therapeutic potential of various MO components in human health. Notably, Agrawal and Mehta studied the utilisation of MO seed kernels to treat bronchial asthma. Their clinical experiment indicated that MO seed extract was safe to deliver to patients, with no side effects reported 110. Stohs and Hartman conducted a review of five human studies on the effects of whole leaf powders of M. oliefera when taken orally. According to the studies, these powders appear to have anti-hyperglycemic, anti-dyslipidemic, and antioxidant properties in humans, without causing severe side effects. These human trials, which used whole leaf powder at doses of up to 50 g and 8 g per day for 40 days, revealed no side effects 111. In a randomised controlled trial by Taweerutchana *et al.*, 25% of the 16 patients who received MO experienced diarrhoea, which resolved on its own within a few days. There were no cases of hypoglycemia, and no significant changes in renal or liver function tests were observed from baseline to end of study 112. Meanwhile, in a clinical trial of 26 pregnant women who ate yoghurt with 4.3 g of dried, ground MO leaves daily, all births were uneventful except for three premature deliveries 113. Furthermore, six randomised controlled trials of MO as a galactagogue in nursing mothers found no adverse effects 114. The variable methods of administration and dosing may have contributed to the varied findings. Currently, most commercially available MO products are not controlled by bodies such as the Food and Drug Administration (FDA) and therefore their exact contents are not known. The systematic review by Popoola *et al.* (covering studies from 1990 to 2019) presents solid evidence of MO's pharmacological multifunctional therapeutic agent with neuroprotection, cardioprotection, anti-inflammatory, and antidiabetic properties. These findings lend credence to the safety profile of MO preparations in people, showing its potential as a treatment for a variety of disorders with minimal toxicity 115. However, more study, including human clinical trials, is required to establish the efficacy, appropriate doses, and long-term safety of MO. There have been no human clinical trials examining the use of MO as a therapy for osteoporosis. This limitation makes it difficult to draw firm conclusions about its efficacy and safety in humans for this specific ailment. However, preclinical evidence (i.e., studies in animals or *in vitro*) indicating osteogenic (bone-forming) actions and anti-osteoporosis qualities could serve as a foundation for future study, including clinical trials.

Cytochrome P-450 (CYP) enzymes are a large family of enzymes involved in the metabolism of a wide range of substances, including drugs 116.* In vitro* studies of MO and cytochrome P-450 (CYP) enzymes revealed that MO may have the ability to suppress some CYP isoenzymes, notably CYP3A4, CYP1A2 and CYP2D6 117, 118. Thus, co-administration of MO with pharmaceuticals that are substrates for certain CYP enzymes may cause substantial drug interactions, affecting treatment efficacy and safety. However, in a trial of HIV-infected patients, the pharmacokinetics of nevirapine, which is a medicine metabolised by CYP3A4, remained unchanged when combined with 1.85 grams of MO powder daily for 14 days 119. The absence of significant interaction with nevirapine in the study suggested no clinically relevant impact on the metabolism of nevirapine.

While these studies provide valuable information, it is crucial to consider that interactions can be drug-specific and context-dependent. Additional studies may be needed to explore interactions with other drugs metabolised by CYP3A4 to confirm the findings in broader populations. Caution is always recommended when combining herbal supplements with prescription medications. Variability in individual responses and differences in product formulations warrant careful consideration and monitoring.

To put MO promising preclinical effects into practice, well-designed human clinical trials are required. These studies would have to assess dose-effect relationships within human-adjusted ranges to verify that the dosages utilised are both effective and safe. Furthermore, the trials must demonstrate that there are no safety risks associated with the use of MO preparations in humans, especially given individual diversity in sensitivity to the plant's bioactive components.

## 7. Conclusion and Perspective

Research on the impact of MO on bone health is on the rise. The objective of this article was to examine the preclinical studies on MO with emphasis on its osteogenic and osteoinductive abilities in preventing or treating bone loss. Preclinical research revealed the potential benefits of MO as functional food, notably for bone health. These results provide the framework for human clinical trials to investigate further into MO's role in reducing osteoporosis risk and enhancing general bone health. If MO is proven to be effective, it can be incorporated into treatment regimens for osteoporosis.

MO is being explored as a potential treatment for osteoporosis owing to its dual role as both an anabolic and antiresorptive agent. It enhanced bone repair by increasing the number of osteoblasts, which are crucial for new bone formation. MO could be highly beneficial in repairing bone defects and supporting osteointegration of dental implants. Its ability to enhance bone regeneration, stimulate osteoblast activity and reduce inflammation makes it a promising candidate for improving bone healing and implant outcomes.

In terms of mechanisms, MO directly modulated the expressions of Runx2 and BMP2 to regulate osteoblast differentiation, while decreasing the RANKL/OPG ratio. It also activated the PI3K/Akt/Foxo1 pathway, which reduced oxidative damage and promoted osteogenic induction. Additionally, MO lowered bone resorption by affecting the expressions of p38α/MAPK14 and RANKL/OPG ratio. These dual effects on osteoblasts and osteoclasts are beneficial for maintaining balance in bone remodelling, which is crucial for managing osteoporosis. Furthermore, MO enhanced the growth, proliferation and differentiation of mesenchymal stem cells into osteoblasts, offering potential advantages for stem cell applications and scaffold-based approaches to improve bone restoration.

## Figures and Tables

**Figure 1:**
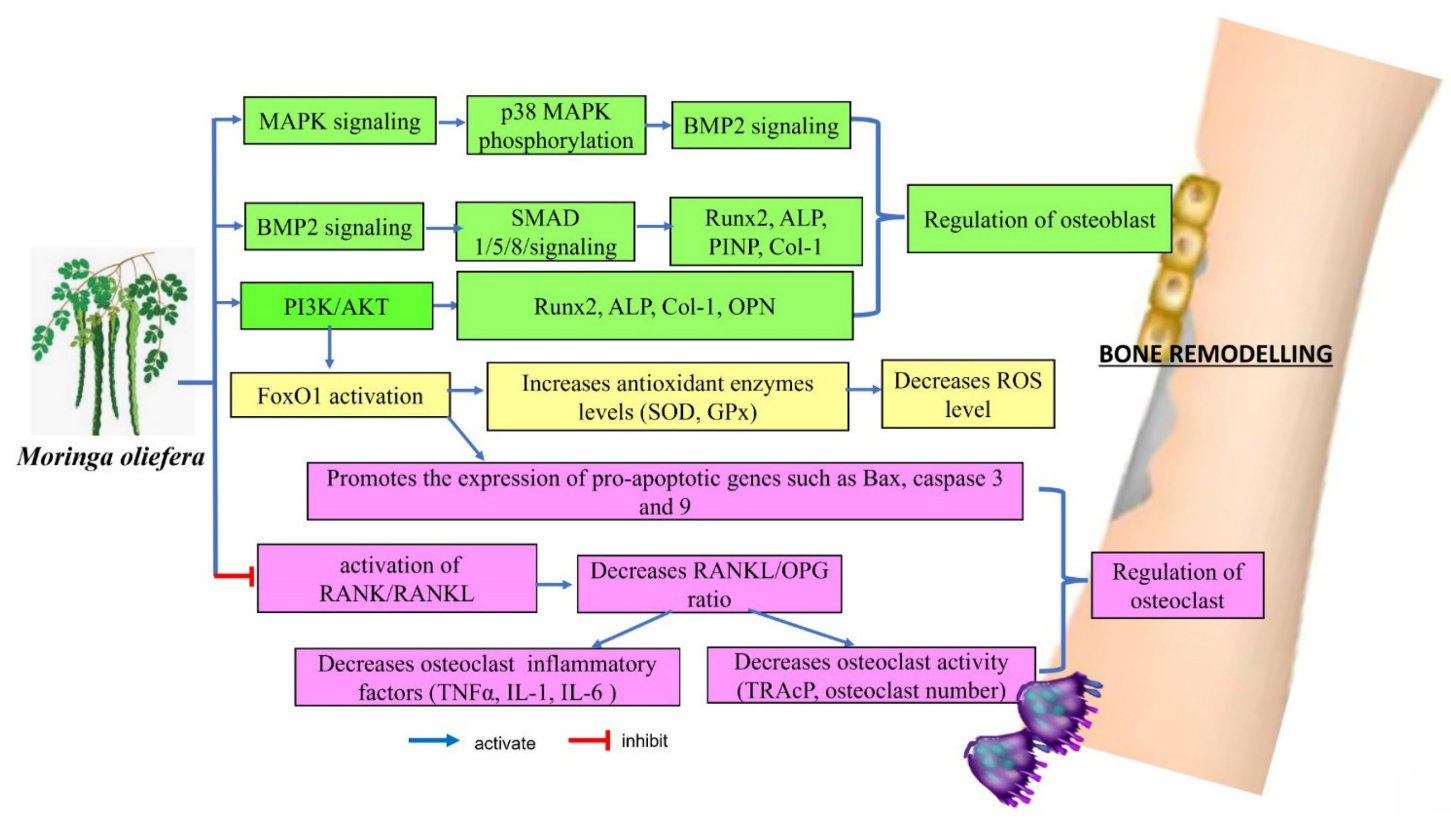
Proposed molecular mechanism of *Moringa oleifera* on bone remodelling.

**Table 1:** Effects of MO on bone in *in vivo* studies. The symbol ↑ denotes an increase, ↓ denotes a decrease, and ↔ denotes no change.

*In vivo* Study	Intervention (Dose, Route, and Duration)	Research Findings	Outcome	References
Female Wistar rats, 3-month-old, bilateral ovariectomy	MO seed oil (0.25 mL/100g BW/day), oral, 60 days	serum Ca ↑, Serum P ↑, ALP ↓, urine Ca ↓, urine P ↓	MO seed oil reduced ALP but increased serum calcium, while decreasing urine calcium and phosphorous levels of ovariectomy rats.	Kusolrat & Kupittayanant 15
Female Sprague-Dawley rats, 10-weeks old, bilateral ovariectomy	MO powder (0.1 mg/kg/day), oral, 12 weeks	BMD ↑, Trabecular: Tb Ar ↑, Tb Sp: ↑, Tb Th ↑, Tb N ↔, Cortical: Ct. Th↑, Ct.Ar ↑, Serum Ca ↑, Serum P ↑, ALP ↑, PINP ↑, Urine Ca ↓ , urine P ↓, CTX-1 ↓, DPD/CREA ↓	MO increased bone mineral density and improved bone metabolism-related indicators and bone microstructure of ovariectomy rats.	Hu *et al.*,^20^
Female Wistar rats, 3-month-old, streptozotocin induced diabetic + ovariectomised (STZ-OVX)	MO leaf, flower and fruit (200mg /100g BW/day), oral, 30 days	ALP ↑, TRAcP↓	MO protected against diabetic and ovariectomy induced osteoporosis by increasing ALP (bone formation marker) and decreasing TRAcP (bone resorption markers)	Patel *et al.*, 26
Albino rats, 3-month-old, dexamethasone (glucocorticoid)-induced jawbone osteoporotic rats	MO leaves (200mg/kg/day), oral, 30 days	jawbone BMD ↑, RANKL ↓, OPG ↑, RANKL/OPG ↓, bone area percentage ↑	MO leaf extract reversed steroid induce osteoporosis by reducing RANKL and increasing OPG expressions, increasing bone area and osteoblast numbers.	Soliman *et al.*,^28^
Albino Sprague Dawley rats, 3-month-old, prednisoneacetate(glucocorticoid)-induced osteoporotic rats	MO leaves, seeds or combination of leaves and seeds (2.5%/kg/day), oral, 8 weeks	BMD ↑, serum Ca ↑, Serum P ↑, T4 ↑, PTH ↓	MO leaves, seeds, and combinations reversed steroid induce osteoporosis by increasing BMD, reducing PTH and increasing serum calcium levels.	Habib *et al.*, 29
Sprague-Dawley rats, 3-month-old, prednisolone (glucocorticoid)-induced osteoporotic rats	MO leaves (200mg/kg/day), oral, 28 days	Bone density ↑, serum Ca ↑, ALP ↑	MO reverses steroid induced osteoporosis by increasing bone density, serum calcium and ALP levels.	Rawat *et al.*, 30
Sprague-Dawley rats, 3-month-old, dexamethasone (glucocorticoid)-induced osteoporotic rats	MO (roots) polysaccharides (6g/kg/day), oral, 28 days	BMD ↑, BV/TV ↑, Tb Th ↑, hexosamine to hydroxyproline ratio ↑	MO polysaccharide may treat femoral head necrosis caused by long-term glucocorticoid use by increasing bone histomorphometric parameters and hexosamine to hydroxyproline ratio.	Zhang *et al.*, 32
Male guinea pigs, alveolar bone sockets after tooth extraction	MO leaves extract + demineralized freeze-dried bone bovine xenograft (DFDBBX), 7 and 30 days	RANKL ↓	MO lowered RANKL expressions of alveolar bone sockets post tooth extraction, which is indicative of bone formation.	Soekobagiono *et al.*, 39
Guinea pigs, alveolar bones after tooth extraction	MO leaves extract + demineralized freeze-dried bone bovine xenograft (DFDBBX), 7 14, and 21 days	RANKL ↓, OPG ↑	Combining MO with DFDDM preserved tooth extraction sockets by increasing OPG and reducing RANKL expressions.	Djais *et al.*,^40^
Adult male New Zealand rabbits, critical-sized bone defects in the edentulous portions of mandibles.	MO leaves extract	↑ the surface area of bone, ↑ the number of osteoblasts	MO leaf extract promotes bone repair in critical-sized defects in the edentulous portions of mandible by increasing bone area and osteoblasts number.	Elsadek *et al.*, 41
Albino rats, defect in femora	Dried seeds of MO + marine collagen, 4 weeks	Number of osteoblasts ↑, Number of osteocytes ↑, ALP ↑, TbN ↑. TbAr ↑	Combination of MO and marine collagen promoted bone healing by increasing bone histomorphometric parameters.	Al-Azzawi and Al-Ghaban 43

Abbreviation: BW: body weight; Ca: Calcium; P: Phosphate; ALP: Alkaline phosphatase; BMD: Bone mineral density; Tb.Ar: Trabecular Area; Tb.Th: Trabecular thickness; Tb.N: Trabecular Number; Ct. Th: Cortical Thickness; Ct. Ar: Cortical Area; BV/TV: bone volume/Total volume; PINP: procollagen I n-terminal propeptide; CTX-1: C-terminal telopeptides type I collagen; DPD/CREA: urinary deoxypyridinoline/creatinine ratio; TRAcP: tartrate-resistant acid phosphatase; RANKL: receptor activator of nuclear factor-κB ligand; OPG: osteoproprotegrin; T4: Thyroxine; PTH: Parathyroid hormone

**Table 2:** Effects of MO on bone in *in vitro/ex vivo* studies. The symbol ↑ denotes an increase, ↓ denotes a decrease, and ↔ denotes no change.

*In vitro*/*Ex vivo* Study	Intervention	Research Findings	Outcome	References
SaOS2 cell line	50 μg/mL to 100 μg/mL MO leaves, flowers and fruits	Cell viability ↑, ALP ↑, hydroxyproline content ↑, Ca↑, Col1 ↑	MO extract from the three plant parts (leaves, flowers and fruits) stimulated osteoblast proliferation, differentiation, and mineralisation.	Patel *et al.* 44
SaOS2 cell line	25 μg/mL and 50 μg/mL MO leaves	Cell proliferation ↑, ALP ↑, Runx2 ↑, BMP2 ↑	Biphasic dose-response effect of MO extract on the development of osteoblast-like SaOS2 cells.	Khan *et al.* 45
	100 μg/mL and 200 μg/mL MO leaves	Cell proliferation↓, ROS ↑, cell cycle arrest		
BMSC cells, exposed to 100 μmol/L H_2_O_2_ and Wortmannin (an inhibitor ofphosphoinositide-3 kinase))	10% MO leaves-containing serum	Cell proliferation ↑, ROS ↓, MDA ↓, Runx2 ↑, SOD↑, GSH ↑, PAkt ↑, FoxO1 ↑, Caspase 3 ↓	MO leaf extract activated the PI3K/Akt/Foxo1 pathway, reducing peroxidative damage and promoting osteogenic induction in rat BMSCs.	Liu *et al.* 50
UMR106 cell line	MO leaves hydrogel	Cell viability ↑, ALP ↑	Titanium disc coated with MO hydrogel and seeded with human MSC showed increased proliferation of osteoblast cells.	Pachimalla *et al.* 56
prototype titanium implants on the human MSC		TEM: Osteoblast proliferation↑, Histomorphometry: Bone implant contact ↑, BV ↑		
RAW 264.7 cell line	1000 µg/ml MO leaves	TNF-α, IL-1β, and IL-6, IL-1Ra and IL-10 ↓, OPG ↑, RANKL ↓, OPG/RANKL↑, p38α MAPK ↓	MO inhibited the expression of inflammatory cytokines and reduced alveolar bone resorption by modulating the expressions of p38α/MAPK14 and OPG/RANKL	Wang *et al.* 59

Abbreviation: ALP: Alkaline phosphatase; Col1-Type 1 Collagen; Runx2: runt-related transcription factor 2; ; BMP2: bone morphogenic protein 2; ROS: Reactive oxygen species; MDA: Malondialdehyde; SOD; Superoxide dismutase; GSH: Glutathione; RANKL: receptor activator of nuclear factor-κB ligand; OPG: osteoprotegerin; FoxO1: Forkhead box O1; PIK/AKT: phosphatidylinositol-3-kinase; TNF-α: tumour necrosis factor-α; IL-1β: interleukin-1β; IL-6: Interleukin-6; IL-1β: Interleukin-1β; IL-1Ra: Interleukin-1Ra; MAPK : mitogen-activated protein kinase; BMSC: bone mesenchymal stem cells; BV: bone volume; TEM: transmission electron microscopy
